# Examining weekly facilitated group sessions and counselor‐crafted self‐monitoring feedback on treatment outcome in digital weight control: A pilot factorial study

**DOI:** 10.1002/osp4.585

**Published:** 2022-01-05

**Authors:** Delia S. West, Rebecca A. Krukowski, Melissa L. Stansbury, Doris Ogden, Janna Borden, Jean R. Harvey

**Affiliations:** ^1^ Department of Exercise Science Arnold School of Public Health University of South Carolina Columbia South Carolina USA; ^2^ Department of Public Health Sciences University of Virginia Charlottesville Virginia USA; ^3^ Department of Nutrition and Food Sciences University of Vermont Burlington Vermont USA

**Keywords:** interactive group sessions, online behavioral weight control, self‐monitoring feedback

## Abstract

**Objective:**

Weight control programs that incorporate group sessions produce greater weight losses, but this has not been explored in the context of online programs. Further, counselor‐crafted self‐monitoring feedback is a core element of lifestyle interventions, although pre‐scripted, modular feedback which does not require detailed counselor review may adequately promote weight loss. The current study explored the weight losses achieved in an online program that included facilitated group sessions, as well as outcomes when counselor‐crafted self‐monitoring feedback was provided.

**Methods:**

A 2 × 2 pilot factorial randomized participants (90% women) with overweight/obesity (*N* = 73) to facilitated group sessions (yes/no) and type of feedback (counselor‐crafted/pre‐scripted, modular) within a 16‐week online behavioral weight control program. Weight change outcomes were collected digitally. Treatment engagement and intervention delivery time were also tracked.

**Results:**

Individuals offered weekly facilitated online group sessions lost more weight (−5.3% ± 4.9%) than those receiving the same digital program without group sessions (−3.1% ± 4.0%; *p* = 0.04). Those receiving group sessions also demonstrated significantly greater treatment engagement. Individuals receiving pre‐scripted, modular feedback lost significantly more weight (−5.3% ± 4.8%) than those receiving the more traditional counselor‐crafted feedback (−3.1% ± 4.1%; *p* = 0.04), but treatment engagement did not differ between conditions. However, interventionist time required to provide feedback was markedly lower for pre‐scripted than counselor‐crafted feedback (1.4 vs. 3.5 h per participant over 16 weeks, respectively, *p* = 0.01).

**Conclusions:**

Incorporating weekly facilitated online group sessions significantly increased weight losses achieved in a digital lifestyle program. Further, pre‐scripted, modular feedback required significantly less staff time than counselor‐crafted feedback without diminishing weight losses. Thus, group sessions and pre‐scripted feedback warrant consideration when designing digital lifestyle programs.

## 1 INTRODUCTION

Previous research has demonstrated that behavioral weight control programs with group sessions produce greater weight losses than the same program delivered individually, both for in‐person^1^ and phone‐delivered programs.^2^ It is notable that the group‐based treatment produced better weight losses even among people who indicated at baseline that they preferred individual treatment.^1^ However, the important question about whether there is an incremental weight loss advantage when group sessions are incorporated into a weight control program has not been explored within the context of online weight control programs.

Other studies demonstrate that fully online group‐based behavioral weight control programs which incorporate weekly structured online group sessions facilitated by an experienced interventionist achieve clinically meaningful weight losses.^3^, ^4^, ^5^, ^6^, ^7^, ^8^ This previous research supports the idea that group sessions may be an important treatment element for online behavioral obesity interventions. However, without research experimentally manipulating the inclusion of online group sessions in a digital program, it remains unclear what weight loss advantages (if any) accrue with group sessions in an online program.

There are notable burdens for both providers and participants when facilitated group sessions are incorporated into online treatment. Thus, this question is not trivial. The challenges associated with scheduling and attending meeting times, as well as staffing costs, can be insurmountable burdens.^9^, ^10^, ^11^ In some areas, finding experienced staff to administer the weight control programs presents an additional hurdle.^12^, ^13^ Therefore, it is important to know whether adding weekly group sessions meaningfully increases weight losses achieved in an online program.

Another key online obesity treatment component for which there are few data to guide online weight control program design is feedback on self‐monitoring. Although behavioral feedback has been established as a critical element to achieve optimal weight loss outcomes in online programs,^14^, ^15^ the nature and extent of feedback necessary to support superior weight losses has not been established. Most online weight control approaches provide some form of feedback on self‐monitoring, usually focused on dietary intake and physical activity.^16^, ^17^ Detailed feedback is a core element of “gold standard” in‐person weight management interventions like the Diabetes Prevention Program^18^ and the Look AHEAD Lifestyle Intervention.^19^ However, the professional time required to review individual weekly self‐monitoring records and provide detailed, personalized feedback can be a principal driver of interventionist time resulting in higher program delivery costs.^20^, ^21^

Although some studies have shown that algorithm‐driven structured feedback on dietary self‐monitoring can produce clinically meaningful weight losses,^14^ few studies have directly compared weight losses achieved with counselor‐crafted personalized feedback on self‐monitoring records with weight loss among individuals who receive pre‐scripted, modular feedback which does not require detailed review by an interventionist. If a modular approach with pre‐scripted feedback proves to be effective in promoting weight loss, the potential scalability and cost savings could be substantial.

Thus, the current study examined the inclusion of weekly group sessions and the type of self‐monitoring feedback incorporated into an evidence‐based online weight control program to evaluate acceptability and feasibility of procedures as well as whether a “signal of effect” emerged with respect to these two critical program design elements. This 2 × 2 pilot factorial design efficiently compared^22^ the main effects for both facilitated group sessions (vs. no group sessions) and counselor‐crafted feedback (vs. pre‐scripted, modular feedback) on weight loss outcomes and treatment engagement. The pilot focused on the main effects of the two treatment components rather than the interaction between them.

## 2 MATERIALS AND METHODS

### 2.1 Study design

This 4‐month pilot study enrolled 73 adults with overweight/obesity in an empirically validated group‐based online weight control program.^3^, ^4^, ^5^ Participants were randomized to receive the program with or without weekly facilitated online group video chat sessions and to one of two types of weekly emailed feedback (pre‐scripted or counselor‐crafted). All participants received the same dynamic online 16‐week online behavioral weight loss program content and strategies. All outcomes were collected digitally; the primary outcome was weight change. Treatment engagement and staff time were also tracked.

### 2.2 Participants

Participants were recruited from across the United States using boosted Facebook posts (paid advertisements that promoted the study's Facebook page to a targeted audience), other non‐paid social media posts and email‐circulated flyers, which directed interested individuals to an online portal that offered a study description and application. Recruitment was conducted from September to October 2020 over 10 weeks. Eligibility criteria included: age 18 years or older; BMI of 27–55 kg/m^2^; no contraindications for participation in a behavioral weight reduction program containing an exercise component; access to a computer (at home or work) with Internet and a video camera, as well as a smartphone; and willingness to provide permission to transmit self‐monitoring and weight data from the Fitbit.com website/app to the study website. The study provided potentially eligible participants with Wi‐Fi enabled scales, which transmitted weight data in real time to study staff. Participants were required to successfully complete seven consecutive days of dietary self‐monitoring as a behavioral run‐in, be available for the day/time that group chats were scheduled (in case they were randomized to participate in online group sessions), and successfully transmit baseline weight on the study‐provided scale to be eligible.

### 2.3 Randomization

Individuals were randomized by a statistician external to the study team within gender using a randomized permutated block sequence. Assignment to group session condition was communicated to participants in an email, which also provided their secure password to access the intervention website; participants were not informed of their feedback assignment. Individuals were aggregated into four “closed” cohorts representing (1) no group sessions/pre‐scripted feedback, (2) no group sessions/counselor feedback, (3) weekly group sessions/counselor feedback and (4) weekly group sessions/pre‐scripted feedback.

### 2.4 Online behavioral weight control program

Participants in all conditions received the same core online group lifestyle intervention, which has been implemented previously.^3^, ^4^, ^5^ The 16‐session, goal‐driven behavioral weight control program was based on social cognitive theory^23^ and used a self‐regulation approach to developing new lifestyle habits to produce and maintain weight loss.^24^ Weekly interactive video modules presented behavioral strategies which support making changes in diet and exercise habits. All participants received the same calorie and physical activity goals and were asked to self‐monitor their dietary intake and physical activity daily using the Fitbit app, which directly transmitted data to the study website. Everyone was also asked to weigh themselves daily on the study‐provided “smart‐scale” (Renpho®). The password‐protected website provided weekly dynamic programmatic content presenting behavioral topics (See Table S1), a discussion bulletin board providing weekly posts to promote asynchronous group interactions related to the weekly topic and engender social support, and educational resources to support behavior change. Each participant had access to personalized online graphic feedback on weight trajectory and physical activity (total steps and minutes of MVPA), which was updated in real‐time. All participants received weekly emailed feedback (pre‐scripted or counselor‐crafted depending upon randomization condition). Each “closed” cohort of 17–20 participants was led by the same interventionist, a dietitian and exercise physiologist with extensive experience facilitating online behavioral weight control; she monitored the discussion board posts for all groups, provided emailed feedback to those in the counselor‐crafted condition and facilitated sessions for those randomized to receive group sessions.

#### 2.4.1 Facilitated group sessions

Weekly synchronous (i.e., in real time) video group sessions (i.e., “face‐to‐face”) were conducted via Zoom.^25^ These counselor facilitated interactions reinforced the information and behavioral strategies introduced in the weekly module and elicited the experiences of participants applying behavioral skills to establish new diet and exercise habits and guided problem‐solving, group cohesion and social support. The 60‐min group sessions followed a protocol with a structured format: Starting with a “check in,” group members reviewed their successes and challenges in meeting dietary, physical activity and other behavioral goals over the previous week and problem‐solved identified barriers. The check‐in was followed by a discussion of the self‐regulation topic introduced in the module for that week, with an emphasis on experiential engagement around the topic and a goal of promoting behavioral skill development. The group session ended with an opportunity for asking questions and a review of the goals for the upcoming week.

#### 2.4.2 Self‐monitoring feedback.

All participants were provided with weekly emailed feedback focused on their self‐monitoring and other weight control behaviors; feedback focused on successful enactment of self‐regulatory behaviors. For those randomized to receive *counselor‐crafted feedback*, the interventionist reviewed dietary, physical activity, and weight monitoring for the week, as well as completion of online modules and group session attendance (for those randomized to weekly group sessions) and composed an individualized feedback message. The emailed feedback provided positive reinforcement for successful goal achievement, identified possible areas for improvement, and suggested possible strategies for identified challenges.^26^ Feedback was crafted using a “Sandwich” approach that bookended praise for successes around constructive suggestions.^10^, ^26^

Individuals randomized to *pre‐scripted, modular feedback* also received a weekly email, but theirs was constructed by selecting from pre‐scripted messages that aligned with success, partial success, or absence of self‐monitoring within each of the following domains: dietary monitoring, physical activity, self‐weighing, and weekly module completion. The pre‐scripted feedback was “modular” in that one of three options was selected. Thus, feedback content was individualized only to the extent that there were pre‐established thresholds to guide selection of the “correct” pre‐scripted message to send that week, so the feedback matched individual performance in each domain. The appropriate pre‐scripted option was selected depending upon the behavior examined. For example, self‐monitoring of dietary intake options reflected monitoring dietary intake and achieving the calorie goals on most days (success), monitoring on most days but not meeting calorie goals over the week (partial success) and not monitoring dietary intake on most days (absence). Feedback on self‐weighing options from which to select included weights on 7 days (success), at least 1 day that week (partial) and no weight submitted (absence). Feedback followed the same structure each week, with headings that identified the domain and then the feedback message (See Table S2 for further details). The modular messages were developed by a clinical psychologist with extensive experience designing and implementing online behavioral weight control programs; a master‐level intervention staff member reviewed the number of self‐monitoring days, calorie intake reported, and modules completed in the study database to guide selection of the correct pre‐scripted message.

### 2.5 Measures

All data were collected electronically, with questionnaires administered via REDCap and body weight data obtained from Wi‐Fi enabled e‐scales provided to study participants, which transmitted data directly to the study website.

#### 2.5.1 Weight

*Change in body weight at 4 months was the primary study outcome* (See Table S3 for further details). Weight change was calculated as kg lost from baseline and % of baseline body weight lost. The proportion of individuals who achieved clinically significant weight losses of ≥5% and ≥10% within each condition was also calculated.

#### 2.5.2 Sociodemographic characteristics

Participants completed online questionnaires with sociodemographic information (i.e., age, gender, race) at baseline.

#### 2.5.3 Intervention engagement

Engagement parameters monitored included number of days of self‐monitoring (weight, dietary intake, and physical activity), completion of the 16 weekly online modules, and attendance at facilitated group sessions (for those randomized to receive them).

#### 2.5.4 Treatment delivery costs

Intervention staff time was tracked throughout the project, including time spent conducting facilitated group sessions, moderating bulletin board postings, and generating feedback. Total time required to deliver these intervention activities was calculated and then averaged across participants within condition to arrive at a time cost for each of experimental factors. Time was tracked rather than costs per se because there is a wide range in the costs associated with staffing intervention delivery across the nation.^11^ Time requirements were considered to be the most informative metric, allowing individual programs to apply locally‐relevant financial costs corresponding to the required intervention time and arrive at accurate estimation of likely economic costs to the provider.

### 2.6 Data analysis

This 2 × 2 factorial was designed to examine the main effects of facilitated group sessions (yes/no) and feedback type (counselor‐crafted/pre‐scripted), with weight change as the primary outcome. Secondary analyses examined treatment engagement and intervention delivery time costs. Missing weight data were imputed with baseline observation carried forward for intent‐to‐treat analyses (ITT), and conditions were compared using *t*‐tests. Analyses of the primary outcome (weight change) were done both as ITT and completers. The nonparametric Mann–Whitney–Wilcoxon test was performed for group comparisons of process measures due to non‐normal distributions. All analyses were conducted using SPSS version 27. Statistical significance was defined as *p* < 0.05.

## 3 RESULTS

### 3.1 Participant characteristics

**FIGURE 1:**
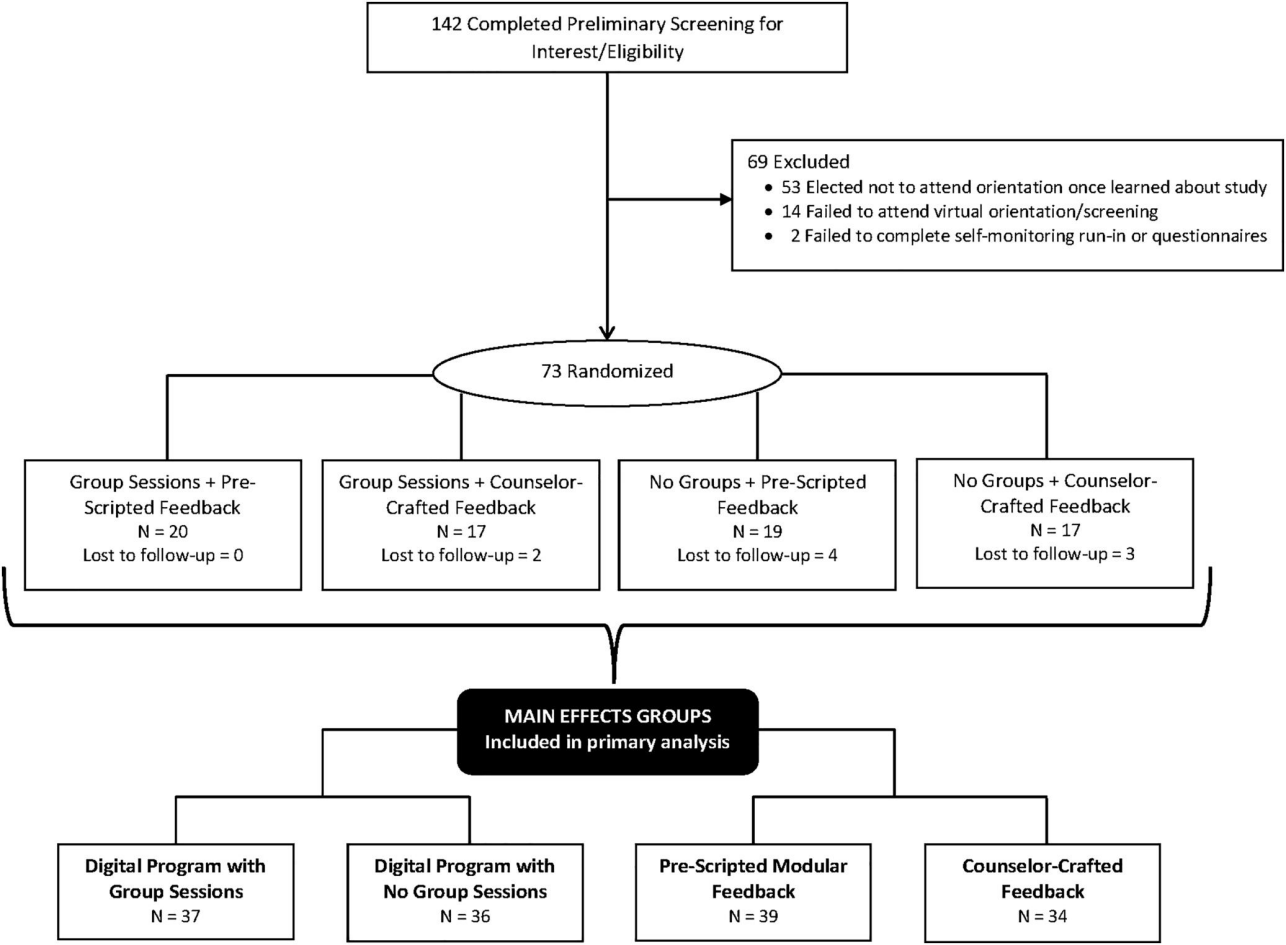
Consort diagram

Participants (*N* = 73) were enrolled and randomized over the course of 10 weeks, with individuals indicating they resided in 20 different states. The majority of participants were women (90%) in their early 50 s who were classified as having obesity, with 16.4% identifying as African American or another racial minority group (Table 1). There were no significant differences across conditions in baseline characteristics for either of the randomization factors (group sessions or feedback type). Further, there was no significant difference in attrition based on either of the randomization factors. Retention rates were high overall, with 88% of participants providing post‐treatment (4‐month) weight data (Figure 1).

**TABLE 1:** Baseline participant characteristics and retention rates by randomization factors

	Sample overall	Group sessions			Feedback type		
		Weekly group sessions	No group sessions	*p‐*value	Pre‐scripted modular feedback	Counselor‐crafted feedback	*p‐*value
*N*	73	37	36		39	34	
Age	50.5 ± 11.2	51.9 ± 11.7	49.1 ± 10.6	0.169	49.6 ± 11.8	51.6 ± 10.4	0.455
Gender (female, %)	90.4%	91.7%	89.2%	1.00	87.2%	51.6%	0.438
Race (%)				0.22			0.12
White	83.6%	89.2%	77.8%		77.0%	91.2%	
Minority^a^	16.4%	10.8%	22.2%		23.1%	8.8%	
Weight (kg)	100.8 ± 20.0	103.8 ± 21.0	97.6 ± 18.8	0.186	103.4 ± 23.1	97.8 ± 15.7	0.269
BMI (kg/m^2^ mean)	35.8 ± 6.1	36.7 ± 6.3	34.8 ± 5.9	0.113	36.4 ± 6.8	35.0 ± 5.3	0.442
Having obesity (BMI ≥ 30, %)	83.6%	86.5%	80.6%	0.49	82.1%	85.3%	0.709
4‐month retention (%)	87.7%	94.6%	80.6%	0.085	89.7%	85.3%	0.725

*Note*: Fisher's exact test for gender and race. Chi‐square test for Having Obesity and 4‐month retention. Mann–Whitney‐*U* test for age, weight, and BMI due to non‐normally distributed data, with the exception of age in analysis comparing structured versus detailed feedback groups (independent *t*‐test conducted). All tests two‐sided at *p* < 0.05 level of significance.

Abbreviation: BMI, body mass index.

^a^ African American (*n* = 10; 13.7%), American Indian or Alaska Native (*n* = 1; 1.4%); Asian (*n* = 1; 1.4%) and Hispanic (*n* = 1; 1.4%).

### 3.2 Weight change

Individuals offered weekly online facilitated group sessions lost more weight than those provided the same online program without group sessions (Table 2). Further, a higher proportion of individuals offered weekly online group sessions achieved ≥10% weight loss at 4‐month than did participants who were not offered group sessions, although there was no significant difference in the proportion of participants losing ≥5%.

**TABLE 2:** Weight loss outcomes at 4 months by treatment condition

	Intent‐to‐treat			Completers only		
	Group sessions included	No group sessions	*p*‐value	Group sessions included	No group sessions	*p*‐value
N included in analysis	37	36		35	29	
Weight loss, kg	5.5 (5.3)	3.1 (4.4)	0.04	5.8 (5.3)	3.8 (4.6)	0.12
Weight loss, %	5.3 (4.9)	3.1 (4.0)	0.04	5.6 (4.8)	3.9 (4.2)	0.14
≥5% weight loss, *n* (%)	16 (43.2%)	10 (27.8%)	0.13	16 (45.7%)	10 (34.5%)	0.26
≥10% weight loss, *n* (%)	7(18.9%)	1 (2.8%)	0.03	7 (20.0%)	1 (3.4%)	0.05

	Pre‐scripted modular feedback	Counselor‐crafted feedback	*p*‐value	Pre‐Scripted modular feedback	Counselor‐crafted feedback	*p*‐value
*N* included in analysis	39	34		35	29	
Weight loss, kg	5.5 (5.5)	2.9 (4.0)	0.02	6.1 (5.4)	3.4 (4.1)	0.03
Weight loss, %	5.3 (4.8)	3.1 (4.1)	0.04	5.9 (4.7)	3.6 (4.3)	0.05
≥5% weight loss, *n* (%)	18 (46.2%)	8 (23.5%)	0.04	18 (51.4%)	8 (27.6%)	0.05
≥10% weight loss, *n* (%)	7 (17.9%)	1 (2.9%)	0.04	7 (20.0%)	1 (3.4%)	0.05

*Note*: Values shown as mean ± SD or frequency counts (percentages) for intent‐to‐treat analyses using baseline observation carried forward for missing data. Independent samples *t*‐test for continuous variables. Fisher's exact test for categorical variables.

Participants receiving pre‐scripted, modular feedback achieved significantly greater weight losses than participants receiving counselor‐crafted, individualized feedback, and a greater number of those receiving pre‐scripted feedback reached the clinically significant milestone weight loss thresholds at 4‐month.

### 3.3 Treatment engagement

Individuals who were offered weekly facilitated group sessions were more engaged in treatment than were those who were not offered group sessions as part of their online program (Table 3). Those who were offered group sessions weighed themselves on significantly more days and self‐monitored dietary intake, physical activity and steps on more days than did those offered the program without weekly group video sessions. There was no significant difference, however, in the number of weekly online modules completed.

**TABLE 3:** Treatment engagement parameters (mean ± SD) by treatment condition

	Group session included			Feedback type		
	Weekly group sessions (*n* = 37)	No group sessions (*n* = 36)	*p‐*value	Pre‐scripted modular feedback (*n* = 39)	Counselor‐crafted feedback (*n* = 34)	*p‐*value
Days of self‐monitoring (out of 111 days)						
Weight	91.8 ± 25.2	72.0 ± 35.2	0.008	85.1 ± 29.8	77.6 ± 34.6	0.07
Dietary intake	77.7 ± 26.9	56.4 ± 35.3	0.005	69.1 ± 32.2	64.7 ± 34.2	0.53
Minutes of moderate‐to‐vigorous physical activity	71.2 ± 32.4	52.2 ± 37.8	0.034	57.4 ± 36.7	66.7 ± 35.6	0.62
Steps taken	98.3 ± 28.1	76.3 ± 37.9	0.007	87.2 ± 35.0	87.4 ± 35.5	0.93
Weekly modules completed (out of 16 weekly modules)	13.3 ± 4.6	10.8 ± 5.9	0.11	12.5 ± 5.0	11.6 ± 5.8	0.39
Weekly group session attendance	12.9 ± 4.4	N/A	N/A	14.4 ± 2.5	11.1 ± 5.5	0.058
Treatment delivery time						
Total interventionist time to deliver (# hours over 16 weeks)	83.6	23.7	0.006	54.8	137.0	0.0001
Hours per participant	2.3	0.7	0.01	1.4	3.5	0.01

*Note*: Values shown as mean ± SD. Mann–Whitney–Wilcoxon test for between‐group comparisons of module completion.

In contrast, treatment engagement did not differ consistently between groups receiving pre‐scripted or counselor‐crafted feedback. Although there were trends toward greater daily self‐weighing and greater attendance at group sessions (for those randomized to weekly group sessions), adherence to self‐monitoring of diet and physical activity was similar between the two feedback groups.

### 3.4 Treatment delivery time costs

Interventionist time to deliver the program with weekly facilitated group sessions totaled 83.6 h over the 4‐months or 5.2 h/week. Lower time costs were associated with delivering the online program without the weekly group sessions (1.5 h/week); time was spent facilitating asynchronous social interaction and answering questions related to the weekly intervention content on the bulletin board. Comparative time costs were 2.3 h per participant in the facilitated group sessions condition over the 16 weeks and 0.7 h per participant in the no group sessions condition.

Interventionist time for providing feedback was markedly lower in the pre‐scripted feedback condition than the counselor‐crafted condition (Table 3). The pre‐scripted approach required approximately 1.4 h per participant over the 16‐week program, compared with 3.5 h per participant for the counselor‐crafted condition.

## 4 DISCUSSION

This study sheds light on two important components of online behavioral obesity treatment programs. The inclusion of “face‐to‐face” (via video) group sessions facilitated by an experienced interventionist increased average weight losses by 2% compared to the same digital weight control program without synchronous group sessions. This suggests that online programs should consider incorporating (or continuing to incorporate) interactive, synchronous group sessions to achieve greater weight loss outcomes. However, pre‐scripted, modular feedback may be adequate for achieving optimum weight losses, and bespoke feedback crafted for individuals by the interventionist may not be necessary.

Including group sessions as part of online lifestyle treatment is not without challenges for both providers and participants, including finding mutually convenient meeting times and staffing group sessions with experienced interventionists. Online delivery of group sessions may reduce some attendance barriers, but scheduling and time availability can still present issues for some participants and identifying qualified intervention staff (and the costs of employing them) can be an impediment for program administrators.^9^, ^10^, ^11^ Despite the challenges, treatment engagement was higher among those offered group sessions and average weight losses exceeded the 5% threshold indicating a clinically meaningful health impact, with 19% of participants offered group sessions achieving ≥10% weight loss at 4‐months. Thus, further exploration of the benefits for including group sessions in online weight control appears warranted.

The mechanism by which group sessions may augment outcomes in digital weight loss is not fully clear. The importance of social support for weight loss success is well‐established.^27^, ^28^ Counselor‐led synchronous interactions may offer critical social support elements which are not obtained from the asynchronous online exchanges (e.g., bulletin board posts). Counselors imposed a theory‐based framework on group interactions that interpreted experiences within a social‐cognitive perspective, prompted problem solving and goal setting when appropriate, and shaped effective weight control behaviors with reinforcing comments, all with the end goal of building self‐efficacy for weight management. Although posting to online bulletin boards can provide social support, it appears that the social interaction provided by real‐time exchanges conferred additional benefits for weight loss.^29^ Alternatively, “face‐to‐face” video interactions with the counselor may have heightened accountability and, thus, increased treatment engagement, which in turn could have augmented weight loss.^30^ Individuals who know they will “see” their counselor at a designated time may feel some positive social pressure to complete self‐monitoring tasks; those without the deadline of a group session may not feel this same accountability to complete the self‐regulatory tasks and, thus, may not lose as much weight. Future studies are needed to parse out the independent and/or complementary contributions of social support and accountability that can accompany group sessions, so that programs can seek to isolate the relevant influences and potentially amplify the effects to accrue additional impact on weight loss outcomes.

Unexpectedly, pre‐scripted modular feedback produced significantly greater weight losses than the more traditional approach of individually tailored, custom‐crafted feedback by a health professional. Those participants provided with pre‐scripted, modular feedback lost significantly more weight than did those provided with feedback that was crafted for the individual, and the professional time (and thus expense) required for generating individual‐specific, counselor‐crafted feedback was substantially greater. Taken together, this suggests that counselor‐crafted feedback may not be warranted. This finding needs to be replicated, but the results provide strong indications that programs could consider adopting a pre‐scripted, modular approach to reduce treatment delivery costs without sacrificing weight loss efficacy.

Others have similarly shown that “more is not necessarily better” when it comes to self‐monitoring feedback,^31^ but the standard within the field remains to have interventionists review and comment on tracking records.^18^, ^19^ Feedback is a central self‐management strategy in behavioral weight control. However, the mechanisms underlying the impact of feedback are poorly defined.^32^ Suggestions that the effects of feedback message content within a digital self‐monitoring app may depend on the self‐monitoring history of the individual^33^ are intriguing and hint that the optimal feedback approach may be dynamic. Further exploration of approaches to providing feedback to optimize weight loss outcomes is warranted; a better understanding of the ideal frequency for feedback (e.g., daily vs. weekly), the way messages are framed (e.g., offering suggestions or advice vs. thought‐provoking questions to prompt self‐reflection on antecedents and consequences of key self‐regulatory behaviors),^33^ and the length of the feedback message^26^ are all important parameters about which little is known.

The study has limitations which must be noted when considering the implications. The sample was small given that it was a pilot study, and consisted predominantly of women, as do most behavioral weight control studies.^34^ However, the sample was national, with broad representation across the country. Further, retention was strong (88%) and, thus, there is limited concern about attrition bias. In addition, the randomized factorial study design is a highly efficient strategy to prospectively determine the contributions of the two treatment strategies^22^ to meaningful weight loss. Nonetheless, we must take care in generalizing the results. Among women seeking online weight control, there are strong signals that the addition of facilitated group sessions may significantly increase weight losses. However, it remains to be seen if men similarly benefit from synchronous groups and whether all‐male groups would be preferable to mixed gender groups. Further, pre‐scripted, modular feedback appears to offer a reasonable alternative to counselor‐crafted individual feedback on self‐monitoring, with significantly lower staff time required to deliver but no negative impact on weight loss outcomes. Finally, this study was implemented early during the COVID‐19 pandemic, when many individuals were sheltering at home and may have had increased time available to participate in a behavioral weight control program and/or experienced weight gain that might have heightened motivation to engage in the program. In addition, disruptions to pre‐COVID patterns of social interaction could have contributed to the impact of the virtual group sessions. Replication of this pilot study outside of these unique circumstances is clearly warranted.

## 5 Conclusions

In this direct comparison of digital behavioral weight control programs offering the same content and asynchronous social support which varied only in the addition of weekly synchronous group sessions, the addition of video group sessions significantly increased weight losses achieved. Therefore, the inclusion of counselor‐facilitated group sessions should be carefully considered in the design of online lifestyle programs and examined in future research. However, the provision of counselor‐crafted self‐monitoring feedback did not confer weight loss benefits over‐and‐above those realized with pre‐scripted, modular feedback, which required substantially less staff time to provide. Thus, a pre‐scripted, modular approach warrants consideration as a method for delivering feedback in a digital lifestyle intervention.

## AUTHOR CONTRIBUTIONS

The authors West, Krukowski and Harvey conceived the study and obtained funding. All authors contributed to implementation of the project, were involved in writing the paper and had final approval of the submitted and published versions

## CONFLICT OF INTEREST

The authors declare no conflicts of interest.

## CLINICAL TRIAL REGISTRATION

CinicalTrials.gov Identifier: NCT04514900.

## Supporting information

Supporting Information S1

Supporting Information S2

Supporting Information S3

Supporting Information S4
