# Supplementary material for: Examining weekly facilitated group sessions and counselor‐crafted self‐monitoring feedback on treatment outcome in digital weight control: A pilot factorial study
Source: Obes Sci Pract. 2022 Jan 5;8(4):433–41. doi: 10.1002/osp4.585 (PMC9358748; doi:10.1002/osp4.585)
Supplement: Supplementary file 1 — Supporting Information S1 [file OSP4-8-433-s002.docx]

| **Supplemental Table 1. iREACH Optimal 16-week Digital Weight Control Program Intervention Modules** | |
| --- | --- |
| **Module/Week** | **Title** |
| 1 | Welcome to iREACH! |
| 2 | Energy in |
| 3 | Energy Out |
| 4 | Goal Setting |
| 5 | Portion Distortion |
| 6 | Lifestyle Activity |
| 7 | Eating Out |
| 8 | Problem Solving |
| 9 | Grocery Guru |
| 10 | Social Support |
| 11 | Party On! |
| 12 | Trip Up Triggers |
| 13 | Automatic Thoughts |
| 14 | Overcome Exercise Hurdles |
| 15 | Don’t Stress Out! |
| 16 | Relapse Prevention & Weight Maintenance |
