# Supplementary material for: Examining weekly facilitated group sessions and counselor‐crafted self‐monitoring feedback on treatment outcome in digital weight control: A pilot factorial study
Source: Obes Sci Pract. 2022 Jan 5;8(4):433–41. doi: 10.1002/osp4.585 (PMC9358748; doi:10.1002/osp4.585)
Supplement: Supplementary file 2 — Supporting Information S2 [file OSP4-8-433-s003.docx]

**Supporting Table 2. Details on Feedback Provided in both the Counselor-crafted Feedback and the Pre-scripted, Modular Feedback Conditions**

**Counselor-Crafted Individual Feedback**

These weekly emails were individually crafted for each participant, allowing the counselor to key in on aspects of the individual’s behavior over the previous week, reference goals or comments made by the participant in monitoring or in the weekly Hone Your Skills activities (interactive skill building activities incorporated in each module, which allowed the counselor to view the individual participant’s responses to queries about goals, challenges, plans for problem solving, etc.). Individuals who failed to self-monitor would be given encouragement to start with at least some days of self-monitoring (or monitoring one aspect of behavior, usually weight). Those who were not viewing the weekly modules would be urged to look at the content, and those who were assigned to receive weekly group sessions who were not attending would be invited to come to the upcoming video chat (and reassured not to worry about coming back after missing a session). For those who were more engaged with the self-monitoring task, reinforcement for establishing a regular pattern of self-weighing, achieving daily calorie goals and/or meeting the weekly physical activity goals would be the focus of the feedback.

Feedback length varied by individual and week of the program. An examination of all feedback sent in the first month of the program within the Counselor-crafted Feedback Condition indicated that the messages averaged 180 words (range: 77-404 words), reflecting the degree of engagement and personalization for each participant. Feedback was emailed weekly via the study website with an email signature that listed the counselor facilitating the group (either the counselor who led the synchronous group sessions for those assigned to receive the weekly video chats in the Group condition or the counselor who facilitated the asynchronous bulletin board social support for those participants assigned to the No Group condition).

**Sample Week 2 Counselor-crafted Feedback Message**

Dear <*Participant Name*>,

As always, so nice to see you in group!  I hope you are having a lovely Thanksgiving season.  I know that many people are having a non-traditional Thanksgiving due to COVID, but I hope that you are still making it a special day.

Excellent job journaling over the week!  Yes, you can average your Cals over the week.  You also asked how to calculate Calories from fat grams.  Since every gram of fat contains 9 Calories, multiply the number of fat grams by 9 and that will give you the number of Calories from fat.

Great job averaging 1275 Cals/day last week and logging nearly 7,000 or more steps on 3 days. You also logged 175 minutes of aerobic exercise, bravo!

I hope you enjoyed watching module 3 on Exercise.  The many benefits of exercise for both weight loss and wellness were listed, as well as strategies for building a healthy and realistic exercise routine.  Since you have lost 5.8 pounds as of today, I'd encourage you to continue aiming for an average of 1300 Cals/day, since this plan appears to be working well for you and a Cal goal of 1500, as originally recommended, may be too high.  We can revisit this at any time.

The first exercise goal is a minimum of 50 minutes of aerobic exercise per week.  You have already exceeded this, great!

In review, the many benefits of exercise include: weight loss, increased muscle mass and metabolism, improved sleep, improved cardiovascular fitness, decreased risk of certain types of cancer, improved emotional state and decreased anxiety, decreases and helps reverse type 2 diabetes, increased energy and self confidence, it's fun, you are a great role model and are able to enjoy the outdoors and nature, either alone, with your pups, family and/or friends.  Lastly, exercise and weight loss may be beneficial for COVID, both in regards to developing symptoms and reducing risk of morbidity.

Have a wonderful Thanksgiving.  Watch portions and enjoy the day.  Perhaps add a new tradition, such as a family walk!

**Protocol for Pre-scripted, Modular Feedback Condition**

Emailed feedback included a structured message about each of the 4 behavioral domains (5 domains for participants randomized to receive the weekly video group session). The specific message selected for a given participant depended upon their performance the previous week, with broad classifications of success, partial success, and no success (or no self-monitoring, if pertinent). Only one “feedback comment” within each domain was selected for a given individual, using a “mix and match” approach that allowed selection of success within one domain and partial success or lack of self-monitoring in another domain. The length of a given modular feedback email message would depend upon the specific combination of domain feedback comments. Feedback would include a common introductory sentence or two which would reference relevant events (e.g., holidays) and/or specific elements of the weekly lesson, goals, or topic to promote a sense of relevance of the feedback content to the intervention process.

The pre-scripted modular feedback email was sent weekly via the study website with an email signature that listed the counselor facilitating the group (either the counselor who led the synchronous group sessions for those assigned to receive the weekly video chats in the Group condition or the counselor who facilitated the asynchronous bulletin board social support for those participants assigned to the No Group condition).

| **Algorithm for Pre-scripted, Modular Feedback**  **Example from Week 3 of the Program** | | | | | |
| --- | --- | --- | --- | --- | --- |
|  | **BEHAVIORAL DOMAIN** | | | | |
|  | Weight Monitoring | Dietary Intake | Physical Activity | Module/Hone Your Skills Completion | **If Group YES:**  Chat Attendance |
| **Success** | Great job weighing yourself daily this week! Doing it even over a holiday is critical. Well done! | You monitored your dietary intake AND stayed within your calorie goals for the week – even with Thanksgiving. Impressive! Think about how you reduced portion sizes, modified foods or substituted healthier options and you’ve got a game plan for the next holidays! | You got in some physical activity even with this being a holiday week. Nice job! Think back about how you were able to do that, so you have some pointers for holidays down the road. | Good work completing the module and doing the Hone Your Skills activity to get started with increasing your physical activity. As you learned in the module, staying active is one of the keys to sustained weight loss. You are on the path! | Great to see you in chat on Tuesday. People who attend group chats tend to lose the most weight, so you are right on track! |
| **Partial Success** | I see you weighed in a couple of days this week but skipped some days as well. Holidays can do that for folks. But try to stay consistent every day even over the holidays to make sure you have your finger on the pulse of which direction your weight is headed! | Looks like you have recorded some of your foods this week, but I can’t really tell if you stayed within your calorie goal. It can be hard to start out with a calorie goal the week of Thanksgiving! So, hop right back into full dietary monitoring and shoot for your calorie goal this week. Even if you got off track last week, the key skill here is getting back on track. Time to practice that! | Holidays can present a challenge for getting in physical activity but also can offer a change in schedule that allows you to sneak in a few minutes to take a walk. Think about how you could plan in some extra activity for the next holiday as well as boost your exercise in this next week | You will want to complete the Hone Your Skills activity at the end of the Week 3 module to see the recommended physical activity to help you lose weight – and perhaps most importantly to keep the weight off. |  |
| **No Success Achieving Goal** |  | Good job monitoring this week. Helpful to have all that information for what you’ve eating over Thanksgiving. But it looks like you went over your calorie limits. Look over your food records to identify things you could eat less of, modify the recipe for, or substitute a healthier option. Then you’re prepared for the next holiday! | Seems like you are getting some activity but not meeting the goals. Holidays can be challenging that way. See if you can “step things up” so that you are burning more calories. | I notice that you have not viewed the Week 3 module this week on Energy Out. Please watch the module to get the scoop on how physical activity contributes to successful weight loss. | Missed seeing you at group on Tuesday. People who attend group chats tend to lose the most weight, so try to attend. If there are barriers to attending chat I should know about, please let me know. |
| **Absence of Self-Monitoring** | I see that you have not weighed yourself this week. I strongly encourage you to weigh yourself each day – not even taking a break for a holiday. You want to guide your weight loss efforts based on the data – so you need to know the numbers! | Not too much recording this week. It may be tempting to skip monitoring on holidays but that’s a time to really figure out how to manage tempting food. So, hop right back into monitoring so you can start to see how you are doing with meeting your calorie goal. | I don’t see any monitoring of your physical activity. Remember, tracking your exercise can help you see where you stand! Start tracking this week! |  |  |

**Sample Week 3 Pre-scripted, Modular Feedback for a hypothetical participant who self-weighed, recorded their dietary intake but went over their calorie limits, met physical activity goals, had not done the weekly module, and was not randomized to the Group condition.**

Dear <*Participant Name*>,

Thanksgiving in the third week of the program may have presented some challenges. Dealing with holidays and a change in routine can often present obstacles but learning how to cope with these challenges is what sustainable weight loss is all about. You know what they say – as long as you learn something from the experience, it is a success. So, what did you learn? What behaviors during the holidays were you proud of and what behaviors do you want to think about changing for the next holiday?

Weight Monitoring

*Great job weighing yourself daily this week! Doing it even over a holiday is critical. Well done!*

Dietary Monitoring

*Good job monitoring this week. Helpful to have all that information for what you’ve eating over Thanksgiving. But it looks like you went over your calorie limits. Look over your food records to identify things you could eat less of, modify the recipe for, or substitute a healthier option. Then you’re prepared for the next holiday!*

Physical Activity

*You got in some physical activity even with this being a holiday week. Nice job! Think back about how you were able to do that, so you have some pointers for holidays down the road.*

Hone Your Skills

*I notice that you have not viewed the Week 3 module this week on Energy Out. Please watch the module to get the scoop on how physical activity contributes to successful weight loss.*
