# Supplementary material for: Examining weekly facilitated group sessions and counselor‐crafted self‐monitoring feedback on treatment outcome in digital weight control: A pilot factorial study
Source: Obes Sci Pract. 2022 Jan 5;8(4):433–41. doi: 10.1002/osp4.585 (PMC9358748; doi:10.1002/osp4.585)
Supplement: Supplementary file 3 — Supporting Information S3 [file OSP4-8-433-s004.docx]

**Supplemental Materials 3. Using e-Scales for Remote Follow-up Data Collection**

Participants were alerted to “Weigh Days,” which were the designated data collection days for everyone in the study. There were monthly “Weigh Days” so that participants were familiar with the process by the time the final post-treatment, 4-month “Weigh Day” arrived.

Participants were asked to obtain an official assessment weight using the procedures outlined in the email message below. A similar email was sent each month. For individuals who failed to weigh themselves on the designated “Weigh Day,” we sent email reminders and would accept a weight up to 7 days after the designated “Weigh Day.” Individuals who alerted us in advance to a situation which would prevent them from providing a weight on the designed “Weigh Day” were also permitted to provide a weight value up to 7 days before the designated day. Thus, we had a 2-week window around the final Month 4 weight ascertainment. Post-treatment 4-month weight data were obtained on 55 randomized participants (75%) on the designated “Weigh Day #4,” and an additional 9 participants (14%) provided weight data during the 2-week data collection window. Thus, 64 individuals (88% of randomized participants) provided final weight data.

*PARTICIPANT EMAIL CONTENT:*

**WEIGH DAY #1**

**Tuesday, December 1st** is your first monthly assessment for the iREACH Optimal program! To complete your assessment:

1. **Weigh yourself on your Renpho**Ò **e-scale first thing in the morning (before breakfast) on December 1st!** Remember to have your RENPHO app open when you step on the scale and check that your weight syncs with your FITBIT account. You will know we have received your weight data when it appears on your iREACH Progress Graph.
2. **Complete your online questionnaires.** The questionnaires ask about your use of weight management strategies and your evaluation of the treatment components. An email with your unique link to the questionnaires will be sent from [iREACH3@mailbox.sc.edu](mailto:iREACH3@mailbox.sc.edu). Please complete this as soon as you receive your link.

Mark your calendar for the remaining 3 monthly assessments, which are scheduled for:

- December 29th
- January 26th
- February 23rd

If you are away from home during any of the four official "Weigh Days," we kindly ask that you take your scale with you... the beauty of portable technology!!
