# Supplementary figures and images for: Examining weekly facilitated group sessions and counselor‐crafted self‐monitoring feedback on treatment outcome in digital weight control: A pilot factorial study
Source: Obes Sci Pract. 2022 Jan 5;8(4):433–41. doi: 10.1002/osp4.585 (PMC9358748; doi:10.1002/osp4.585)

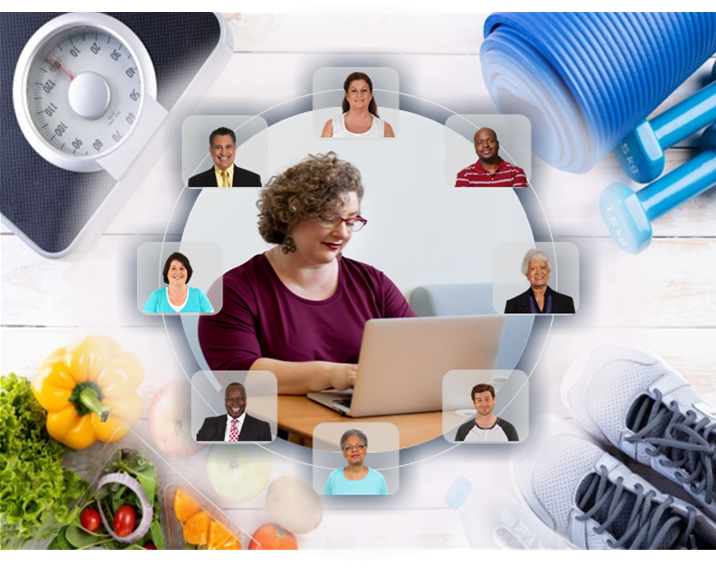

Supplement: Supplementary file 4 — Supporting Information S4 [file OSP4-8-433-s001.png]
